# Characterization of Anthocyanins and Their Antioxidant Activities in Indian Rose Varieties (*Rosa* × *hybrida*) Using HPLC

**DOI:** 10.3390/antiox11102032

**Published:** 2022-10-14

**Authors:** Poonam Kumari, D. V. S. Raju, K. V. Prasad, Supradip Saha, Sapna Panwar, Surinder Paul, Namita Banyal, Aarti Bains, Prince Chawla, Melinda Fogarasi, Szabolcs Fogarasi

**Affiliations:** 1 ICAR-Indian Agricultural Research Institute, New Delhi 110012, India; 2 Division of Agrotechnology, CSIR-Institute of Himalayan Bioresource Technology, Palampur 176061, Himachal Pradesh, India; 3 ICAR-Directorate of Floricultural Research, Pune 411005, Maharashtra, India; 4 ICAR-National Bureau of Agriculturally Important Microorganisms, Kushmaur, Maunath Bhanjan 275101, Uttar Pradesh, India; 5 ICAR-Indian Grassland and Fodder Research Institute, Himachal Pasturelands, Palampur 176061, Himachal Pradesh, India; 6 Department of Microbiology, Lovely Professional University, Phagwara 144411, Punjab, India; 7 Department of Food Technology and Nutrition, Lovely Professional University, Phagwara 144411, Punjab, India; 8 Department of Food Engineering, University of Agricultural Sciences and Veterinary Medicine of Cluj Napoca, Calea Mănăstur 3–5, 400372 Cluj-Napoca, Romania; 9 Department of Chemical Engineering, Faculty of Chemistry and Chemical Engineering, Babeş-Bolyai University, 11 Arany Janos Street, 400028 Cluj-Napoca, Romania; 10 Interdisciplinary Research Institute on Bio-Nano-Sciences, Babeş-Bolyai University, 42 Treboniu Laurian Street, 400271 Cluj-Napoca, Romania

**Keywords:** *Rosa* × *hybrida*, anthocyanins, antioxidant activity, HPLC, nutraceuticals

## Abstract

The present study was designed to explore the anthocyanin profile and antioxidant activities in Indian rose varieties (*Rosa* × *hybrida*). Among fifty varieties, Ashwini recorded the highest total phenolic content (427.59 ± 3.47 mg GAE/100 g) along with the highest FRAP (397.15 ± 0.82 µmol trolox/g) and DPPH free radical scavenging activity (93.47 ± 0.19%) on a fresh weight basis. A significant positive correlation was observed between total anthocyanin content, total phenolic content, and antioxidant activities. Four distinct clusters were formed according to total anthocyanins, total phenols, and antioxidant activities; white- and yellow-colored varieties were most distant from red ones. Principal component analysis revealed that variable total anthocyanin content contributed to the maximum variation among the fifty rose varieties studied. Highly anthocyanin-rich rose varieties were characterized by high-performance liquid chromatography coupled with a photodiode array detector (HPLC-PAD), which identified two major components of anthocyanins, i.e., cyanidin 3,5-di-*O*-glucoside and pelargonidin 3,5-di-*O*-glucoside. Cyanidin 3,5-di-*O*-glucoside was the predominant anthocyanin in red- and pink-colored varieties, whereas pelargonidin 3,5-di-*O*-glucoside was the major one in the orange variety. The maximum cyanidin 3,5-di-*O*-glucoside content was recorded in variety Ashwini (497.79 mg/100 g), whereas the maximum pelargonidin 3,5-di-*O*-glucoside content was recorded in Suryakiran (185.43 mg/100 g). It is suggested that the rose varieties with high anthocyanin content and antioxidant activity can be exploited as a potential source of nutraceuticals in the food industry.

## 1. Introduction

Rose (*Rosa* × *hybrida*) is one of the most stunning flowers of the Rosaceae family, comprising more than 200 species, which are widely distributed throughout the Northern Hemisphere [1,2]. It has a wide variety of color spectra, ranging from delicate whites, yellows, and pinks to strong purple, orange, and red tones, primarily due to the presence of anthocyanin pigments [3,4]. The flower is also well known for its palatable nature and has been devoured for a long time in teas, cakes, and flavor extracts, as well as being a traditional remedy to treat blood circulation conditions and control cancer growth [5,6,7]. Anthocyanins, a significant constituent of the characteristic food colorants belonging to the flavonoid family, are a group of water-soluble pigments that are structurally composed of an aglycone anthocyanidin and sugar moieties. They are responsible for the attractive colors of different fruits, blossoms, and vegetables; moreover, their explicit tone depends upon co-pigments, metal ions, and pH [8,9]. To date, more than 635 anthocyanins have been distinguished, including six common aglycones (cyanidin, peonidin, pelargonidin, malvidin, delphinidin, and petunidin). Anthocyanins do not only provide nature with striking colors; they also have various health-beneficial effects. Previous reports have shown that anthocyanins have high radical scavenging potential and play a vital role in the prevention of cardiovascular diseases, obesity, cancer, diabetes, and other health problems [10]. The phenolic hydroxyl groups in the molecule are responsible for the high antioxidant activity of anthocyanins. The phenolic hydroxyl group can effectively preclude peroxidation by providing a hydrogen atom that can remove free radicals and therefore break down the oxidation chain reaction. Anthocyanins are safe and nontoxic pigments [11]. Various studies have indicated that anthocyanins from rose petals exhibit potent anti-inflammatory, antioxidant, anticancer, antimicrobial, and antiallergic properties that can be utilized in functional foods and cosmetics [6,12,13,14,15,16]. Research on anthocyanins has attracted the utmost attention in recent years, particularly their isolation and purification in some fruits and vegetable crops. In contrast, there have been few studies on flower crops that are well known for the presence of a diverse range of pigments with myriad colors [17]. The potential of anthocyanin pigments from Indian rose varieties, however, is not completely understood. In particular, to the best of our knowledge, former studies have not extensively investigated the anthocyanin profiles and antioxidant activities in Indian rose varieties. Hence, this study was undertaken to profile the anthocyanins and antioxidant activities in rose varieties. The main aim of this study was to search for effective Indian rose varieties having not only high anthocyanin content but also various biological properties. In this paper, we report for the first time the anthocyanin compositions from the petals of Indian edible roses using high-performance liquid chromatography coupled with a photodiode array detector (HPLC-PAD). We also determined individual content in the anthocyanin extracts, as well as antioxidant activity.

## 2. Materials and Methods

### 2.1. Plant Material

**Figure 1:**
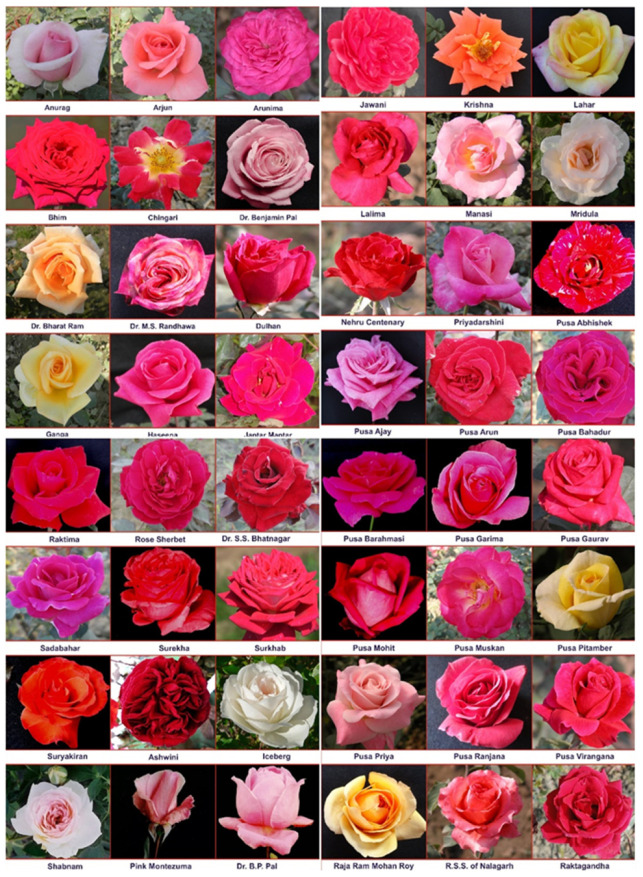
Variability in the flower color of rose varieties used in the present study.

Fresh rose petals of fifty rose varieties were collected in the morning from 8:00 to 9:00 a.m. from the Research Farm of the Division of Floriculture and Landscaping, ICAR Indian Agricultural Research Institute, New Delhi, during December 2015-16. It is located at latitude 28°38′23″ N, longitude 77°09′27″ E, and altitude 228.61m. Soils are sandy loams with a pH of 7.5-7.9. Forty-nine Indian varieties, namely Pusa Arun, Raktima, Nehru Centenary, Pusa Bahadur, Lalima, Bhim, Ashwini, Dr. S.S. Bhatnagar, Jantar Mantar, Jawani, Raktagandha, Pusa Mohit, Pusa Ajay, Pusa Muskan, Pusa Barahmasi, Pusa Virangana, Pusa Priya, Dr. Bharat Ram, Dr. M. S. Randhawa, Priyadarshini, Dulhan, Dr. B.P. Pal, Anurag, Arjun, Haseena, Raja Surendra Singh of Nalagarh, Sadabahar, Nurjehan, Pink Montezuma, Surekha, Dr. Benjamin Pal, Pusa Ranjana, Arunima, Manasi, Rose Sherbet, Pusa Garima, Pusa Gaurav, Suryakiran, Kiran, Shola, Pusa Pitamber, Lahar, Ganga, Raja Ram Mohan Roy, Pusa Abhishek, Mridula, Chingari, Shabnam, Surkhab, and one exotic variety, viz., Iceberg, were included in the present study (Figure 1 and Table 1). All these varieties were well distinguished from each other on the basis of the Distinctness, Uniformity, and Stability (DUS) testing provided by the Protection of Plant Varieties and Farmers’ Rights Authority (PPV&FRA) India.

### 2.2. Chemicals

Anthocyanin standards (cyanidin 3,5-di-*O*-glucoside and pelargonidin 3,5-di-*O*-glucoside), DPPH (2,2-Diphenyl-1-picrylhydrazyl), and TPTZ (2,4,6-Tris(2-pyridyl)-s-triazine) were obtained from Sigma-Aldrich, St. Louis, Missouri, USA. Methanol and acetonitrile for high-performance liquid chromatography coupled with photodiode array detector (HPLC- PAD) were of chromatographic grade, and all the other reagents were of analytical grade.

### 2.3. Histological Studies of Anthocyanin Pigments in Rose

Fifty varieties of rose were screened for total anthocyanin content in two different seasons [4]. Thirteen varieties with high anthocyanin content from red, pink, and orange color groups (Ashwini, Dr. S.S. Bhatnagar, Rose Sherbet, Bhim, Pusa Ajay, Nehru Centenary, Suryakiran, Pusa Arun, Raktima, Raktagandha, Pusa Virangana, Pusa Bahadur, and Surkhab) were analyzed histologically for the distribution of anthocyanin pigments. Cross-sections of rose petals of different varieties were examined using a Carl Zeiss Discovery v8 Stereo microscope (Carl Zeiss Microimaging GmbH, Berlin, Germany) and images were captured with a Carl Zeiss Axiovision digital camera (software version: Axiovision 4.8.2).

### 2.4. Determination of Total Phenolic Content (TPC)

Total phenolic content was assessed according to the procedure given by Singleton and Rossi [18], with slight modifications. A 0.5 g fresh flower sample was extracted with 20 mL 80% methanol. An aliquot (1 mL) of the extract was taken in a test tube and 2.9 mL of Folin and Ciocalteau’s Phenol Reagent (1N) was added, followed by the addition of 0.5 mL of distilled water, and all the tubes were shaken well. Then, 2 mL of sodium carbonate (20%) solution was added to all the tubes and incubated at room temperature for 30 min in the dark. The color developed was read in a spectrophotometer at 750 nm wavelength. The total phenolic content was expressed as mg of gallic acid equivalent (GAE) per gram.

### 2.5. Determination of Antioxidant Activities

#### 2.5.1. Sample Extraction

Each 0.5 g fresh flower sample was extracted with 20 mL of 80% ethanol. The extract was centrifuged at 10,000 rpm at 4 °C for 20 min. The supernatant was taken for determination of total antioxidant activity by FRAP (Ferric Reducing Antioxidant Potential) and DPPH (2, 2-Diphenyl-1-picrylhydrazyl).

#### 2.5.2. Ferric Reducing Antioxidant Potential (FRAP)

The antioxidant activity of rose petals was estimated using the FRAP (Ferric Reducing Antioxidant Potential) method as described by Benzie and Strain [19]. FRAP reagent was prepared by mixing 300 mM acetate buffer pH 3.6, 10 mL TPTZ in 40 mM HCl, TPTZ (2, 4, 6-tripyridyl-s- triazine), and 20 mM ferric chloride (FeCl_3_.6H_2_O) in the ratio of 10:1:1, which was freshly prepared on the same day. The ethanolic extract (0.1 mL) of the flower sample was taken in a test tube and 3 mL of working FRAP reagent was added. Then, the tubes were kept for incubation at room temperature for 4-5 min. The blue color thus developed was read in a spectrophotometer at 593 nm, using the FRAP reagent as a blank, and expressed as μmol of Trolox equivalents per gram of sample. The standard curve was prepared using FeSO_4_ as standard. Different concentrations of FeSO_4_ were prepared and optical density was read at 593 nm. Based on the standard curve, concentrations of samples were calculated.

#### 2.5.3. DPPH Free Radical Scavenging Activity

This method is based on the reduction of DPPH, a stable free radical. The antioxidant activity of rose petal extracts was estimated using the DPPH assay described by Braca et al. [20]. Ethanolic extract (0.1 mL) of the sample was added to 3.9 mL of 0.0025 M DPPH (2, 2-Diphenyl-1-picrylhydrazyl) in methanol (70%). The mixture was shaken and kept for 30 min in the dark at room temperature. Absorbance was recorded at 517 nm in a spectrophotometer. The percentage inhibition of activity was calculated by the following formula:

Percent inhibition (%) = [(Ao − Ae)/Ao] × 100

(Ao = absorbance without extract; Ae = absorbance with extract).

### 2.6. HPLC Analysis of Anthocyanin Composition of Rose

**Figure 2:**
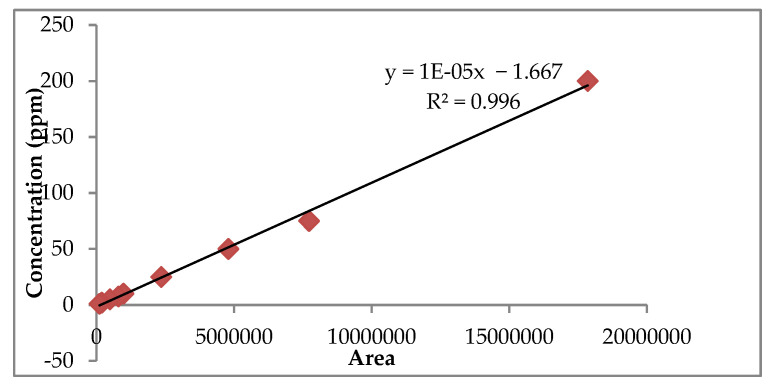
Calibration curve for standard cyanidin 3,5-di-*O*-glucoside.

**Figure 3:**
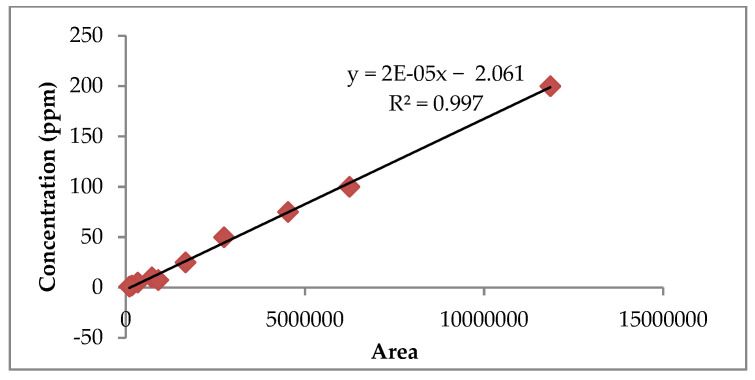
Calibration curve for standard pelargonidin 3,5-di-*O*-glucoside.

Fifty varieties of rose were screened for total anthocyanin content in two different seasons by Kumari et al. [4]. Varieties with high anthocyanin content from red, pink, and orange color groups (Ashwini, Pusa Arun, Nehru Centenary, Dr. S.S. Bhatnagar, Raktima, Raktagandha, Pusa Bahadur, Bhim, Suryakiran, Pusa Virangana, Rose Sherbet, Surkhab, and Pusa Ajay) were systematically characterized by high-performance liquid chromatography (HPLC) coupled with a photodiode array detector for their anthocyanin composition. In the morning, flowers of the above-mentioned varieties were plucked and the petals were carefully removed (2 g) and taken into an amber flask, and extracted with 500 mL of acidified methanol (0.1% HCl). The content was sonicated in the dark for 30 min on an ultrasonicator (Misonix, NY, USA) and the extract was concentrated under a vacuum (35 ± 1 °C) in a rotary evaporator (Heidolph, Germany) for the complete removal of methanol. HPLC-grade methanol was added to the dried sample. Prior to HPLC analysis, supernatant was filtered through a 0.45 µm syringe filter (Whatman Inc.; Maidstone, UK). The anthocyanin content was quantified based on the modified procedure described by Zheng et al. [21]. The peak area of the anthocyanin was integrated from the HPLC chromatogram at 520 nm using the Agilent ChemStation software and plotted against concentration. The stock solutions were made with 1% TFA (*v*/*v*) in methanol to give a 1 µg/mL concentration. The calibration curve was obtained for cyanidin-3,5 di-*O*-glucoside and pelargonidin 3,5 di-*O*-glucoside at 5 different concentrations (50, 100, 150, 200, and 250 ppm), and each sample’s absorbance was measured at 520 nm so as to obtain the following linear equations, Y = 1E-05X-1.667 (r^2^ = 0.9967) and Y = 2E-05X-2.061 (r^2^ = 0.997), respectively (Figure 2 and Figure 3). The anthocyanin content was finally calculated according to the standard curve and expressed as mg/100 g on a fresh weight basis. The purity of anthocyanin powder concentrate was checked with the HPLC instrument using a mobile phase comprising a gradient mixer of solvent A: water (0.1% TFA) and solvent B: water: ACN: TFA (53:46:1 *v*/*v*) at a flow rate of 0.6 mL min-1. The gradient mobile phase was A: 80% for 0 min, 40% in the next 26 min, 80% for 14 min, and the total run time was 40 min. The chromatogram was acquired at 520 nm after injection of 20 µL. Standard cyanidin-3,5 di-*O*-glucoside and pelargonidin-3,5 di-*O*-glucoside were also run according to the above-mentioned flow rate. Visible spectra were also recorded for each peak found in the HPLC analysis. Peak identification was done according to retention time, elution order, and spectra obtained as compared with standards under the same conditions. The concentration of identified anthocyanins was calculated based on cyanidin-3,5 di-*O*-glucoside and pelargonidin-3,5 di-*O*-glucoside equivalent, whereas unknown anthocyanins were quantified as cyanidin 3-*O*-glucoside.

### 2.7. Statistical Analysis

All assays were performed in triplicate. All the data were subjected to Turkey’s honestly significant difference (HSD) test for the comparison of means, and significance for the test was assumed if *p* ≤ 0.05. The analyses were carried out through the statistical software SPSS 20.0 (SPSS Inc.; Chicago, IL, USA). Correlation analyses were performed to estimate the relationship between anthocyanin content, antioxidant activities, and phenolic content. Cluster analysis was performed using SAS v9.4 [22]. A principal component analysis (PCA) biplot was constructed using R software.

## 3. Results and Discussion

### 3.1. Histological Studies of Anthocyanin Pigments in Rose

**Figure 4:**
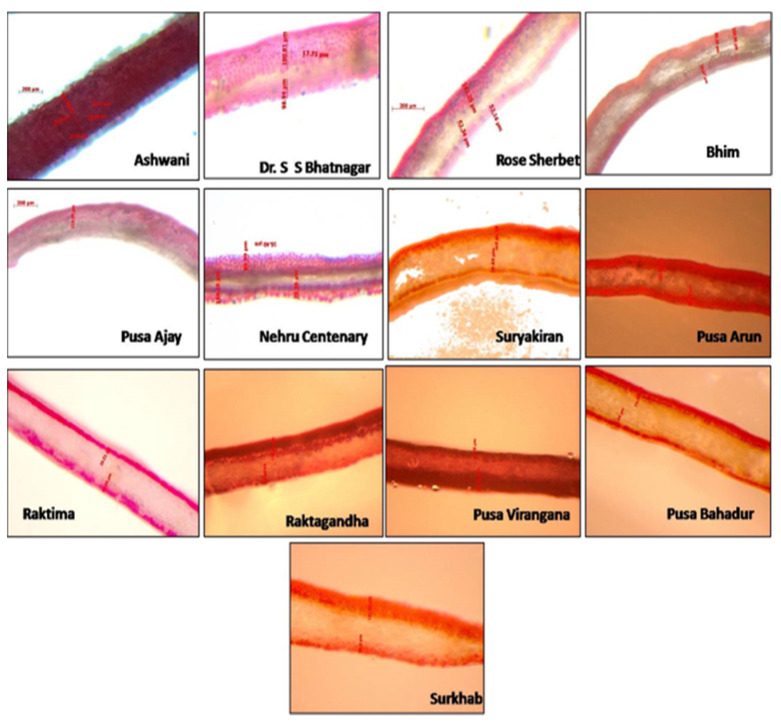
Histological examination of rose petals for anthocyanin distribution.

Histological studies were conducted in varieties Ashwini, Dr. S.S. Bhatnagar, Rose Sherbet, Bhim, Pusa Ajay, Nehru Centenary, Suryakiran, Pusa Arun, Raktima, Raktagandha, Pusa Virangana, Pusa Bahadur, and Surkhab to document the location and distribution of anthocyanin pigments in different cell layers of rose petals (Figure 4). There was variability in the distribution of anthocyanin in the epidermal cell layers of all the studied varieties. It was observed that anthocyanin pigments were distributed in both adaxial and abaxial epidermal cells in all varieties except Pusa Ajay. In addition to adaxial and abaxial epidermal cells, varieties such as Ashwini, Dr. S.S. Bhatnagar, and Pusa Arun also produced pigments in subepidermal cells and mesophyll cells. This may be the reason that these varieties were found superior in terms of their total anthocyanin content. Pusa Ajay is a light pink-colored rose variety wherein only the abaxial epidermal layer reported the presence of anthocyanin pigments. In the variety Pusa Virangana, the maximum distribution of anthocyanin pigments was found in the adaxial epidermis as compared to the abaxial epidermis. The relative location of anthocyanin pigments may be as important as the relative concentration in determining the flower color. The result of our study is supported by [23] Mudalige et al. [23]. They investigated the pigment distribution in *Dendrobium* orchids and reported that the color intensity of orchids is determined by the spatial distribution of pigments in different layers of epidermal cells.

### 3.2. Total Phenolic Content

Among 50 rose varieties included in the study, the total phenolic content varied from 5.21 ± 0.39 mg GAE/100 g fresh weight to 427.59 ± 3.47 mg GAE/100 g fresh weight of petals (Table 2). The findings showed that the varieties of light colors exhibited less phenolic content, while the varieties with bright red colors exhibited greater phenolic content in the petals. Previous studies by Zheng et al. [24] also reported that the total phenolic content was found to be the maximum in red- and pink-colored rose species, except for white rose. Qin and Xiaojun [11] reported a total phenolic content of 2087.43 ± 17.37 mg gallic acid equivalents (GAE) per 100 g fresh weight (FW) in rose petals, and another study by Roman et al. [25] also reported that the total phenolic content in *Rosa canina* varied from 326 mg/100 g frozen pulp to 575 mg/100 g frozen pulp.

### 3.3. Antioxidant Activity

The study revealed a significant difference in the antioxidant activities of the diverse genotypes (Table 2). FRAP values among rose varieties ranged from 8.92 ± 0.36 µmol Trolox/g fresh weight (Iceberg) to 397.15 ± 0.82 µmol rolox/g (Ashwini) on a fresh weight basis. The DPPH value among rose varieties ranged from 4.45 ± 0.30% (Iceberg) to 93.47 ± 0.19% (Ashwini). It is evident from the study that high antioxidant activity was observed in dark red varieties, followed by deep pink, orange, pink, yellow, and white. Our results are in close conformity with the findings of Sadighara et al. [26] in *Althaea officinalis*. They reported that reddish-pink flowers of *Althaea officinalis* have higher antioxidant activity than pink and white flowers. The variety Ashwini was found best for anthocyanin content as well as antioxidant activities, which showed that the flower color plays a role not only in the content of anthocyanins but also in antioxidant power. Specifically, a red color is generally associated with high values of antioxidant activity and a white color with the lowest ones. The possible reason for this variation in the antioxidant content of rose varieties may be due to the genotypic effect. Suzan and Sezai [27] concluded that antioxidant activity was influenced by genotype in *Rosa* taxa. Sayed et al. [28] investigated the antiradical activity of flowers of fresh Taif rose by the DPPH method and reported high antiradical activity with SC50 = 49.44 µg/mL. Qin and Xiaojun [11] also investigated the antioxidant activities of the rose by DPPH assay and found that the DPPH radical scavenging activity value was 2089 mg GAE/100 g fresh weight. Our results are also in line with the findings of Zeng et al. [29]. They evaluated extracts from 19 Chinese edible flowers for antioxidant effects. The results showed that the extracts of *Paeonia suffruticosa, Paeonia lactiflora,* and *Rosa rugosa* possessed stronger DPPH radical scavenging activity (94.221 ± 0.102; 93.739 ± 0.424, and 94.244 ± 0.163%, respectively).

### 3.4. Correlation between Total Anthocyanin Content, Total Phenolic Content, and Antioxidant Activities

A strong, statistically significant, positive correlation was observed between the total anthocyanin content, total phenolic content, and antioxidant activity of rose petals (Table 3). The strong correlation between these parameters indicates that varieties with high anthocyanin and phenolic content constitute a good index for antioxidant activities. Our result is in agreement with Nowak and Gawlik-Dziki [30]. They reported a strong, positive correlation coefficient (r = 0.8485) between the DPPH radical scavenging activity and phenolic content of rose. Özgen et al. [31] reported that the total anthocyanin content of *Sambucus canadensis* is correlated to antioxidant capacity values (r = 0.70-0.85). Similarly, Căta et al. [32] also observed in berries that antioxidant activity is directly proportional to anthocyanin content.

### 3.5. Cluster Analysis

**Figure 5:**
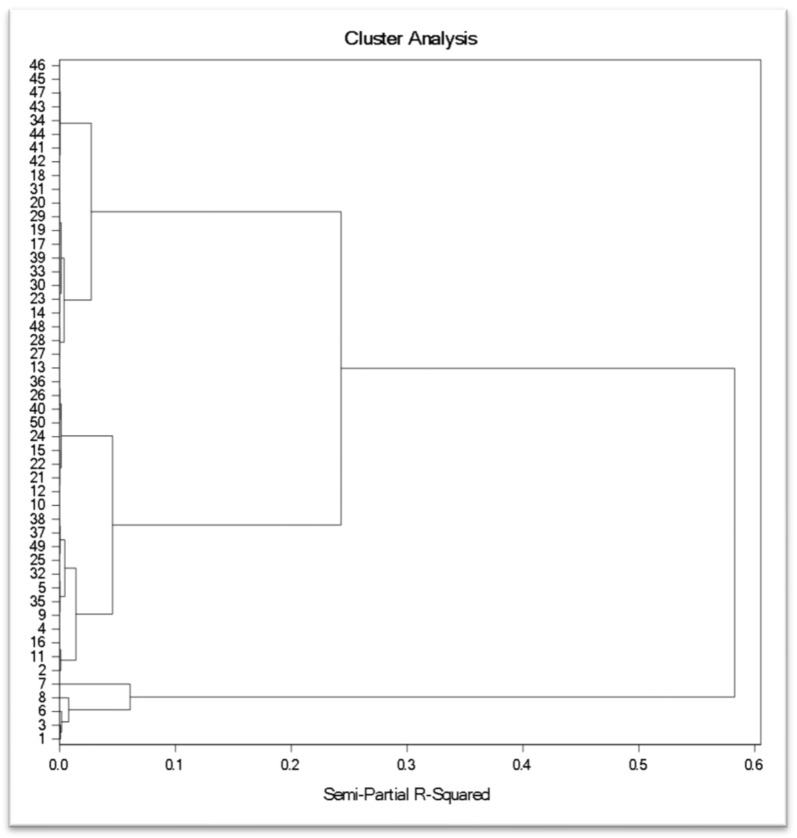
Cluster analysis of different rose varieties. 1: Pusa Arun, 2: Raktima, 3: Nehru Centenary, 4: Pusa Bahadur, 5: Lalima, 6: Bhim, 7: Ashwini, 8: Dr. S.S. Bhatnagar, 9: Jantar Mantar, 10: Jawani, 11: Raktagandha, 12: Pusa Mohit, 13: Pusa Ajay, 14: Pusa Muskan, 15: Pusa Barahmasi, 16: Pusa Virangana, 17: Pusa Priya, 18: Dr. Bharat Ram, 19: Dr. M.S. Randhawa, 20: Priyadarshini, 21: Dulhan, 22: Dr. B.P. Pal, 23: Anurag, 24: Arjun, 25: Haseena, 26: R.S.S. of Nalagarh, 27: Sadabahar, 28: Nurjehan, 29: Pink Montezuma, 30: Surekha, 31: Dr. Benjamin Pal, 32: Pusa Ranjana, 33: Arunima, 34: Manasi, 35: Rose Sherbet, 36: Pusa Garima, 37: Pusa Gaurav, 38: Suryakiran, 39: Krishna, 40: Shola, 41: Pusa Pitamber, 42: Lahar, 43: Ganga, 44: Raja Ram Mohan Roy, 45: Iceberg, 46: Mridula, 47: Shabnam, 48: Chingari, 49: Surkhab, 50: Pusa Abhishek.

Based on Ward’s minimum variance cluster analysis, the fifty rose genotypes were grouped into four clusters based on total anthocyanin content, antioxidant activities, and total phenolic content (Figure 5). Cluster I comprised five dark-red-colored rose varieties, i.e., Pusa Arun, Raktima, Nehru Centenary, Bhim, and Dr. S. S. Bhatnagar. Cluster II comprised 21 rose varieties, i.e., Pusa Bahadur, Lalima, Jantar Mantar, Jawani, Raktagandha, Pusa Mohit, Pusa Barahmasi, Pusa Virangana, Dulhan, Dr. B.P. Pal, Arjun, Haseena, R. S. S. of Nalagarh, Pusa Ranjana, Rose Sherbet, Pusa Garima, Pusa Gaurav, Suryakiran, Shola, Surkhab, Pusa Abhishek. In cluster III, only a single dark-red-colored variety, Ashwini, was placed more divergently. This variety was found superior in terms of total anthocyanin content, with high antioxidant activities. Cluster IV formed the largest cluster, which comprised twenty-three light pink, white, and yellow-colored rose varieties, namely Pusa Ajay, Pusa Muskan, Pusa Priya, Dr. Bharat Ram, Dr. M.S. Randhawa, Priyadarshini, Anurag, Sadabahar, Nurjehan, Pink Montezuma, Surekha, Dr. Benjamin Pal, Arunima, Manasi, Krishna, Pusa Pitamber, Lahar, Ganga, Raja Ram Mohan Roy, Iceberg, Mridula, Shabnam, and Chingari. Rose varieties with high anthocyanin content, antioxidant activities, and total phenolic content were placed in a separate cluster. From the dendrogram, it was also evident that dark-colored varieties were clustered together. Schmitzer et al. [33] also performed cluster analysis of forty-eight cultivars of rose based on total anthocyanins and quercetins and reported that white cultivars, identical to pink and light red cultivars, were the most distant from red cultivars. Singh et al. [34] indicated that crossing between genotypes that are profoundly genetically distinct provides better results as such crosses increase the possibility of obtaining desirable transgressive segregants. The most divergent genotype in the present study is Ashwini, based on cluster distance, and can therefore be selected as a parent for crossing to further enhance the anthocyanin content.

### 3.6. Principal Component Analysis

**Figure 6:**
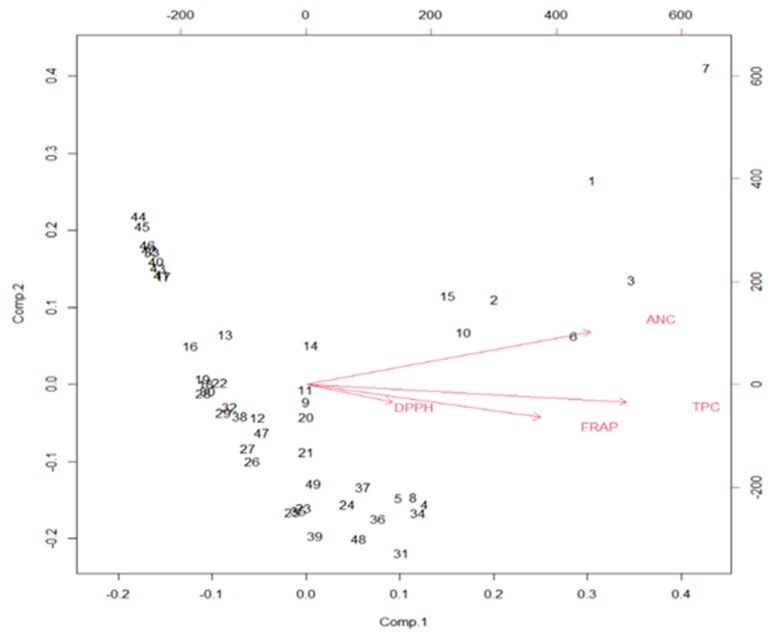
Principal component analysis biplot for total anthocyanin content (ANC), total phenolic content (TPC), and antioxidant activities using FRAP and DPPH methods. 1: Pusa Arun, 2: Raktima, 3: Nehru Centenary, 4: Pusa Bahadur, 5: Lalima, 6: Bhim, 7: Ashwini, 8: Dr. S.S. Bhatnagar, 9: Jantar Mantar, 10: Jawani, 11: Raktagandha, 12: Pusa Mohit, 13: Pusa Ajay, 14: Pusa Muskan, 15: Pusa Barahmasi, 16: Pusa Virangana, 17: Pusa Priya, 18: Dr. Bharat Ram, 19: Dr. M.S. Randhawa, 20: Priyadarshini, 21: Dulhan, 22: Dr. B.P. Pal, 23: Anurag, 24: Arjun, 25: Haseena, 26: R.S.S. of Nalagarh, 27: Sadabahar, 28: Nurjehan, 29: Pink Montezuma, 30: Surekha, 31: Dr. Benjamin Pal, 32: Pusa Ranjana, 33: Arunima, 34: Manasi, 35: Rose Sherbet, 36: Pusa Garima, 37: Pusa Gaurav, 38: Suryakiran, 39: Krishna, 40: Shola, 41: Pusa Pitamber, 42: Lahar, 43: Ganga, 44: Raja Ram Mohan Roy, 45: Iceberg, 46: Mridula, 47: Shabnam, 48: Chingari, 49: Surkhab, 50: Pusa Abhishek.

In this study, a PCA biplot was constructed to determine the contribution and correlation of variables. Total anthocyanin content contributed to the maximum variation among the fifty genotypes studied (Figure 6). Further, it is evident from the biplot that total phenolic content had a strong correlation with antioxidant activity (FRAP). Meanwhile, variety Ashwini was situated in the upper-right quadrant, with the longest distance from the origin, showing that Ashwini had higher total anthocyanin content, total phenolic content, and antioxidant activity (FRAP and DPPH).

### 3.7. Anthocyanin Identification and Characterization

**Figure 7:**
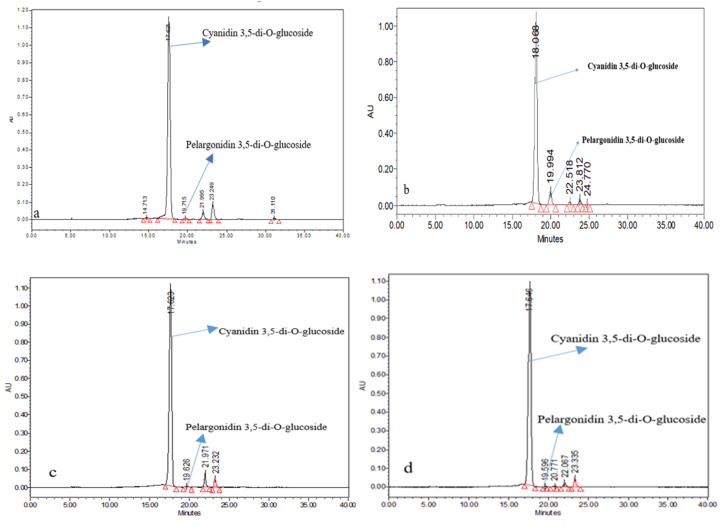

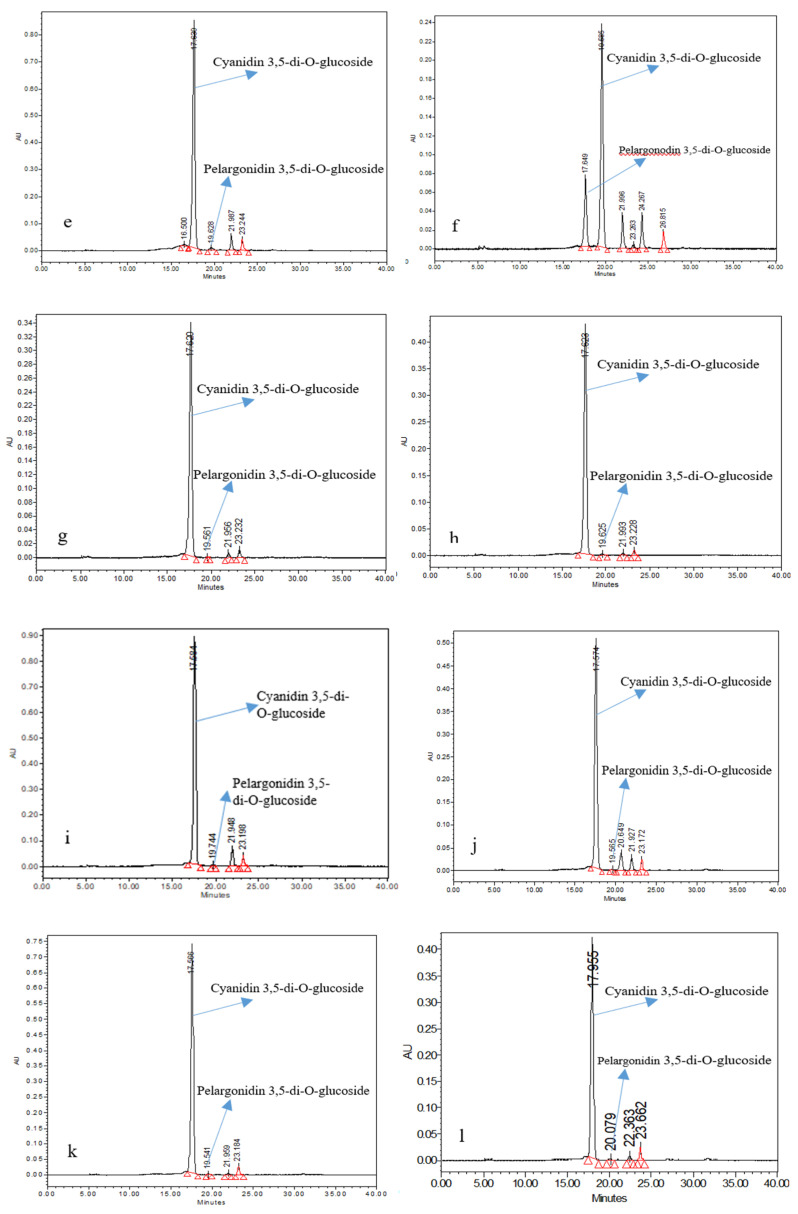

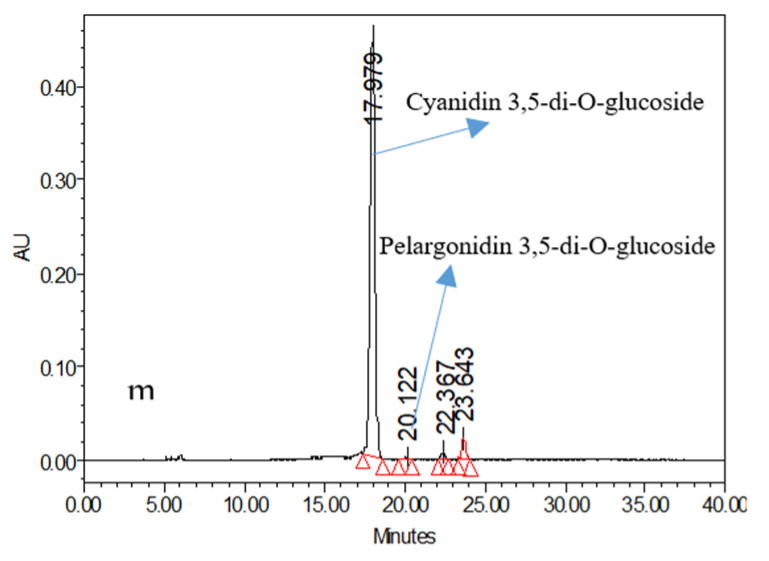
HPLC chromatograms of promising rose varieties. (**a**) Ashwini; (**b**) Pusa Arun; (**c**) Nehru Centenary; (**d**) Raktagandha; (**e**) Raktima; (**f**) Suryakiran; (**g**) Pusa Bahadur; (**h**) Bhim; (**i**) Dr. S.S. Bhatnagar; (**j**) Rose Sherbet; (**k**) Pusa Virangana; (**l**) Surkhab; (**m**) Pusa Ajay.

Several peaks were detected in all varieties, corresponding to different types of anthocyanin fractions. Among all the rose varieties, cyanidin 3,5-di-*O*-glucoside and pelargonidin 3,5-di-*O*-glucoside were the major anthocyanins identified according to retention time, elution order, and spectra obtained by comparison with external standards and published data (Figure 7). In the HPLC analysis, the individual content of different anthocyanin fractions corresponding to different peaks was estimated. Five types of anthocyanins were detected in the variety of Ashwini. Peak 1 and peak 2 had retention times (RT) of 17.621 and 19.715 min, respectively, which was in line with the standards of cyanidin 3,5-di-*O*-glucoside and pelargonidin 3,5-di-*O*-glucoside. In the chromatogram of Ashwini, peak 1 corresponded to cyanidin 3,5-di-*O*-glucoside (497.79 mg/100 g), and peak 2 corresponded to pelargonidin 3,5-di-*O*-glucoside (7.60 mg/100 g); peak 3, 4, and 5 corresponded to unknown anthocyanins, which were expressed as C3G equivalents (17.24, 32.42, and 3.76 mg/100 g, respectively). Cyanidin 3,5-di-*O*-glucoside was the predominant constituent, which accounted for 89.38% of the total anthocyanins according to the analysis results of high-performance liquid chromatography with photodiode array detection (Table 3 and Figure 7a). In the chromatogram of Pusa Arun, peak 1 represented cyanidin 3,5-di-*O*-glucoside (448.54 mg/100 g, RT 18.068 min), peak 2 represented pelargonidin 3,5-di-*O*-glucoside (61.75 mg/100 g, RT 19.994 min), and peak 3, 4, and 5 corresponded to unknown anthocyanins, which were expressed as C3G equivalents (5.88, 10.71, and 1.02 mg/100 g, respectively). Cyanidin 3,5-di-*O*-glucoside was the principal anthocyanin in the variety Pusa Arun, representing 90.24% of the total peak area (Table 3 and Figure 7b). Four types of anthocyanins were observed in the variety Nehru Centenary (Figure 7c). In the chromatogram of Nehru Centenary, peak 1 corresponded to cyanidin 3,5-di-*O*-glucoside (452.12 mg/100 g), which accounted for 91.42 % of the total peak area; peak 2 corresponded to pelargonidin 3,5-di-*O*-glucoside (5.43 mg/100 g), and peak 3 and 4 corresponded to unknown anthocyanins, which were expressed as C3G equivalents (23.10 and 15.74 mg/100 g, respectively). From the chromatogram of the variety Raktagandha (Table 3 and Figure 7d), five anthocyanins were revealed, with peak 1 representing 94.65% of the total peak area. Peak 1 represented cyanidin 3,5-di-*O*-glucoside (457.66 mg/100 g), which was the predominant anthocyanin in the variety Raktagandha. Peak 2 corresponded to pelargonidin 3,5-di-*O*-glucoside (1.31 mg/100 g), and peaks 3, 4, and 5 corresponded to unknown anthocyanins, which were expressed as C3G equivalents (1.31, 7.48, and 16.42 mg/100 g, respectively). Four types of anthocyanins were observed in the variety Raktima (Figure 7e). The maximum peak area was found under peak 2 (89.20 %), which represented cyanidin 3,5-di-*O*-glucoside (338.91 mg/100 g, RT 17.639 min); peak 3 corresponded to pelargonidin 3,5-di-*O*-glucoside (10.66 mg/100 g, RT 19.628 min); peak 3 and 4 corresponded to unknown anthocyanins, which were expressed as C3G equivalents (17.33 and 13.77 mg/100 g, respectively). From the chromatogram of variety Suryakiran, six anthocyanins were revealed, with peak 2 representing 60.83% of the total peak area. Peak 1 represented cyanidin 3,5-di-*O*-glucoside (29.28 mg/100 g, RT17.649). Peak 2 corresponded to pelargonidin 3,5-di-*O*-glucoside (185.43 mg/100 g, RT 19.585 min), which was the prime anthocyanin in variety Suryakiran (Figure 7f). The chromatogram of Pusa Bahadur revealed four types of anthocyanin (Figure 7g). Peak 1 corresponded to cyanidin 3,5-di-*O*-glucoside (137.59 mg/100 g), which represented 95.62% of the total peak area. Peak 2 represented pelargonidin 3,5-di-*O*-glucoside (0.39 mg/100 g). Four types of anthocyanins were observed in the variety of Bhim (Figure 7h). In the chromatogram of Bhim, peak 1 corresponded to cyanidin 3,5-di-*O*-glucoside (175.80 mg/100 g, RT 17.623 min), which occupied 96.72% of the total peak area. Peak 2 corresponded to pelargonidin 3, 5-di-*O*-glucoside (2.31 mg/100 g, RT 19.625 min), and peaks 3 and 4 corresponded to unknown anthocyanins, which were expressed as C3G equivalents (1.50 and 3.32 mg/100 g, respectively). From the chromatogram of variety Dr. S.S. Bhatnagar (Figure 7i), four anthocyanins were revealed, with peak 1 representing 90.78% of the total peak area, which corresponded to cyanidin 3,5-di-*O*-glucoside (362.40 mg/100 g), and peak 2 corresponded to pelargonidin 3,5-di-*O*-glucoside (1.98 mg/100 g). The chromatogram of the variety of Rose Sherbet revealed five types of anthocyanins (Figure 7j). Peak 1 corresponded to cyanidin 3,5-di-*O*-glucoside (206.57 mg/100 g), which was the chief anthocyanin in Rose Sherbet, with 86.35% of the total peak area. As shown in Figure 7k, four anthocyanins were revealed in the variety of Pusa Virangana, with peak 1 representing cyanidin 3,5-di-*O*-glucoside (301.66 mg/100 g). Four types of anthocyanins were observed in the variety Surkhab, among which cyanidin 3,5-di-*O*-glucoside (173.60 mg/100 g), which occupied 93.63% of the total peak area, was the predominant one (Figure 7l). In variety Pusa Ajay, peak 1 corresponded to cyanidin 3,5-di-*O*-glucoside (190.05 mg/100 g), which represented 94.08% of the total peak area (Figure 7m). Statistically significant differences were observed for cyanidin 3,5-di-*O*-glucoside and pelargonidin 3,5-di-*O*-glucoside content among all varieties (Table 3). The maximum cyanidin 3,5-di-*O*-glucoside content was recorded in Ashwini (497.79 mg/100 g), followed by the variety Raktagandha (457.66 mg/100 g). The cyanidin 3,5-di-*O*-glucoside content of Nehru Centenary (452.12 mg/100 g) and Pusa Arun (448.54 mg/100 g) was statistically similar. The lowest cyanidin 3,5-di-*O*-glucoside content was recorded in the variety Suryakiran (29.28 mg/100 g). Pelargonidin 3,5-di-*O*-glucoside content also showed significant variation among all varieties. The maximum pelargonidin 3,5-di-*O*-glucoside content was recorded in variety Suryakiran (185.43 mg/100 g), followed by Pusa Arun (61.75 mg/100 g). According to Eugster et al. [35] the anthocyanin types of rose petals depend upon the plant variety, being mostly cyanidin 3,5-di-*O*-glucoside, pelargonidin 3,5-di-*O*-glucoside, and peonidin 3,5-di-*O*-glucoside. In our study, cyanidin 3,5-di-*O*-glucoside content was the maximum in red- and pink-colored varieties, whereas Suryakiran is an orange color variety and recorded the highest pelargonidin 3,5-di-*O*-glucoside content. Our results are well supported by the findings of Ludmila et al. [36]. They evaluated red-, pink-, and orange-colored varieties of rose and reported the cyanidin 3,5-di-*O*-glucoside and pelargonidin 3,5-di-*O*-glucoside content. They concluded that pink- and red-colored varieties had more cyanidin 3,5-di-*O*-glucoside content, whereas the orange-colored varieties were rich in pelargonidin 3,5-di-*O*-glucoside.

Factors including genetics, environmental stresses, variety, and agronomic conditions can influence the types and content of anthocyanin pigment [37,38]. Our findings suggest that cyanidin 3,5-di-*O*-glucoside and pelargonidin 3,5-di-*O*-glucoside were the most predominant anthocyanins found in Indian roses using reversed-phase C18 column chromatography. These results are similar to the findings of Lee et al. [12] and Wan et al. [39], who characterized anthocyanins from the rose as cyanidin 3,5-di-*O*-glucoside and pelargonidin 3,5-di-*O*-glucoside. The most predominant anthocyanin was cyanidin 3,5-di-*O*-glucoside in all the varieties (Figure 7). These results are in accordance with a previous study reporting that cyanidin 3,5-di-*O*-glucoside was identified as the main component in rose varieties [40]. The findings of Qin and Xiaojun [11] are also in line with our results as they detected three types of anthocyanins in rose petals, and, based on the results of high-performance liquid chromatography with photodiode array detection, cyanidin 3,5-di-*O*-glucoside was the prime constituent and represented 94.9 percent of the total anthocyanins. Biolley et al. [41] found cyanidin 3,5-diglucoside and pelargonidin 3,5-diglucoside in 100 cyanic cultivars of *Rosa* x *hybrida* using the HPLC technique.

## 4. Conclusions

A significant positive correlation was observed between total anthocyanin content, total phenolic content, and antioxidant activities. Principal component analysis revealed that variable total anthocyanin content contributed to the maximum variation among the fifty rose varieties. A significant difference was observed in the anthocyanin profile among the rose varieties. This research presented a systematic report on the anthocyanin composition in the petals of 13 distinct color varieties. Two anthocyanins were identified in the Indian varieties of rose with the help of standards. Cyanidin 3,5-di-*O*-glucoside was the predominant anthocyanin in red- and pink-colored varieties, whereas pelargonidin 3,5-di-*O*-glucoside was predominant in orange-colored varieties. Anthocyanin content can be increased through a breeding program based on crosses between the most divergent genotypes. This study provides a basis for rose breeding to achieve a specific flower color. Further, the rose varieties with high anthocyanin content may be utilized as sources of anthocyanins, which have immense applications in the nutraceutical and food industries as food colorants, antioxidants, and anticancer, antimicrobial, and antiallergic agents, promoting health and wellbeing.

## Figures and Tables

**Table 1:** List of rose varieties along with their parentage, group, and color.

Sr. No.	Cultivar Name	Parentage	Group	Color
1	Pusa Arun	Queen Elizabeth x Jantar Mantar	Hybrid Tea	Dark red
2	Raktima	Hybrid seedling of Pink Parfait x Sugandha	Hybrid Tea	Red
3	Nehru Centenary	Christian Dior x Avon	Hybrid Tea	Dark red
4	Pusa Bahadur	Cara Mia x Century Two	Hybrid Tea	Red
5	Lalima	Picture x Jour d’ete	Hybrid Tea	Red
6	Bhim	Charles Mallerin x Delhi Princess	Hybrid Tea	Red
7	Ashwini	Not Known	Hybrid Tea	Dark red
8	Dr. S.S. Bhatnagar	Not Known	Floribunda	Dark red
9	Jantar Mantar	Not Known	Hybrid Tea	Red
10	Jawani	Samourai x Louisiana	Hybrid Tea	Red
11	Raktaghandha	Christian Dior x seedling of Carrousel	Hybrid Tea	Red
12	Pusa Mohit	Suchitra x Christian Dior	Hybrid Tea	Pink
13	Pusa Ajay	Pink Parfait x Queen Elizabeth	Hybrid Tea	Pink
14	Pusa Muskan	Pink Parfait x Alinka	Floribunda	Color blend (Cream with pink edges)
15	Pusa Barahmasi	Not Known	Floribunda	Pink
16	Pusa Virangana	Selection from an open-pollinated seedling of Jantar Mantar	Floribunda	Red
17	Pusa Priya	Jantar Mantar x Queen Elizabeth	Hybrid Tea	Pink
18	Dr. Bharat Ram	Sweet Afton x Ganga	Hybrid Tea	Pink
19	Dr. M.S. Randhawa	Sabine x Kiss of Fire	Hybrid Tea	Color blend (White and pink)
20	Priyadarshini	Pink Parfait x First Prize	Hybrid Tea	Color blend (White and pink)
21	Dulhan	Bonne Nuit x Ena-Harkness	Hybrid Tea	Red
22	Dr. B.P. Pal	Not Known	Hybrid Tea	Pink
23	Anurag	Sweet Afton x Gulzar	Hybrid Tea	Pink
24	Arjun	Blithe Spirit x Montezuma	Hybrid Tea	Pink
25	Haseena	Youki San x Balinese	Hybrid Tea	Pink
26	Raja Surendra Singh of Nalagarh	Samourai x Montezuma	Hybrid Tea	Pink
27	Pusa Sadabahar	Seedling of Frolic	Floribunda	Pink
28	Nurjehan	Sweet Afton x Crimson Glory	Hybrid Tea	Pink
29	Pink Montezuma	Not known	Hybrid Tea	Pink
30	Surekha	Seedling of Queen Elizabeth	Hybrid Tea	Pink
31	Dr. Benzamin Pal	Sweet Afton x First Prize	Hybrid Tea	Pink
32	Pusa Ranjana	Seedling of Sabine	Hybrid Tea	Deep pink
33	Arunima	Seedling of Frolic	Floribunda	Pink
34	Manasi	Not known	Floribunda	Pinkish white
35	Rose Sherbet	Seedling of Gruss an Teplitz	Floribunda	Deep pink
36	Pusa Garima	Not known	Hybrid Tea	Pink
37	Pusa Gaurav	Not known	Hybrid Tea	Deep pink
38	Suryakiran	Not known	Floribunda	Orange
39	Krishna	Not known	Floribunda	Orange
40	Shola	Not known	Floribunda	Orange
41	Pusa Pitamber	Not known	Floribunda	Yellow
42	Lahar	Hybrid seedling of Pink Parfait x Ganga	Floribunda	Yellow
43	Ganga	Seedling of Sabine	Hybrid Tea	Pale yellow
44	Raja Ram Mohan Roy	Not known	Hybrid Tea	Yellow
45	Iceberg	Not known	Floribunda	White
46	Mridula	Queen Elizabeth x Seedling of Sir Henry Segrave	Hybrid Tea	White
47	Shabnam	Seedling of Baby Sylvia	Floribunda	White
48	Chingari	Charleston x Not known	Floribunda	Bicolored (Yellow+ red)
49	Surkhab	Not known	Hybrid Tea	Bicolored (Red + white)
50	Pusa Abhishek	Bud sport of Jantar Mantar	Floribunda	Striped (Pink with white stripes)

**Table 2:** Variation in total phenolic content and antioxidant activities of rose varieties.

Varieties	Total Phenolic Content (mgGAE/100 g)	FRAP (μmol Trolox/g)	DPPH (%)
Pusa Arun	306.78 ± 2.46	237.145 ± 0.84	87.43 ± 0.0.10 (69.20)
Raktima	251.71 ± 1.56	192.1467 ± 1.38	83.88 ± 0.05 (66.30)
Nehru Centenary	342.67 ± 3.05	268.31 ± 1.82	89.67 ± 0.16 (71.22)
Pusa Bahadur	219.11 ± 1.67	178.08 ± 0.91	75.62 ± 0.18 (60.39)
Lalima	195.45 ± 3.79	169.83 ± 0.79	71.58 ± 0.16 (57.76)
Bhim	333.20 ± 1.43	216.89 ± 0.78	85.77 ± 0.14 (67.81)
Ashwini	427.59 ± 3.47	397.15 ± 0.82	93.47 ± 0.19 (75.17)
Dr. S.S. Bhatnagar	379.24 ± 2.26	286.97 ± 1.37	91.36 ± 0.05 (72.88)
Jantar Mantar	209.12 ± 0.86	174.22 ± 1.09	72.62 ± 0.17 (58.43)
Jawani	132.38 ± 3.75	102.27 ± 1.08	62.73 ± 0.08 (52.34)
Raktagandha	217.99 ± 3.84	193.32 ± 4.31	81.80 ± 0.10 (64.72)
Pusa Mohit	124.21 ± 2.05	106.22 ± 0.81	64.35 ± 0.16 (53.32)
Pusa Ajay	101.03 ± 2.32	86.76 ± 1.70	35.71 ± 0.18 (36.68)
Pusa Muskan	67.18 ± 1.95	64.68 ± 1.69	33.61 ± 0.17 (35.40)
Pusa Barahmasi	116.45 ± 1.76	110.81 ± 0.96	66.44 ± 0.12(54.58)
Pusa Virangana	201.22 ± 1.37	183.93 ± 1.28	80.67 ± 0.08(63.90)
Pusa Priya	44.77 ± 2.68	51.11 ± 1.09	20.74 ± 0.05 (27.08)
Dr. Bharat Ram	21.77 ± 0.59	29.59 ± 0.62	15.74 ± 0.16 (23.36)
Dr. M.S. Randhawa	55.78 ± 2.65	65.37 ± 0.86	23.88 ± 0.00 (29.24)
Priyadarshini	55.17 ± 2.43	61.23 ± 1.12	22.63 ± 0.10 (28.40)
Dulhan	124.10 ± 2.85	114.32 ± 2.66	58.49 ± 0.09 (49.87)
Dr. B.P. Pal	128.45 ± 1.39	116.07 ± 1.32	59.45 ± 0.10 (50.43)
Anurag	64.44 ± 0.90	72.35 ± 0.71	28.52 ± 0.08 (32.27)
Arjun	131.06 ± 0.90	121.78 ± 00.73	57.42 ± 0.06 (49.25)
Haseena	163.04 ± 1.96	142.60 ± 1.18	60.41 ± 0.08 (50.99)
R.S.S. of Nalagarh	129.69 ± 2.15	113.97 ± 1.99	46.41 ± 0.12 (42.93)
Sadabahar	94.56 ± 1.22	96.05 ± 0.71	31.47 ± 0.13 (34.11)
Nurjehan	90.71 ± 0.61	92.25 ± 0.54	30.69 ± 0.09 (33.63)
Pink Montezuma	54.74 ± 2.12	65.49 ± 0.82	21.52 ± 0.15 (27.63)
Surekha	70.68 ± 0.54	77.31 ± 0.59	25.86 ± 0.05 (30.56)
Dr. Benjamin Pal	61.76 ± 1.78	63.94 ± 0.50	22.76 ± 0.11(28.49)
Pusa Ranjana	199.61 ± 0.65	179.70 ± 1.92	70.47 ± 0.12 (57.06)
Arunima	69.94 ± 3.32	83.10 ± 1.03	28.48 ± 0.13 (32.24)
Manasi	12.21 ± 0.97	21.92 ± 0.89	10.74 ± 0.10 (19.12)
Rose Sherbet	205.05 ± 3.15	186.23 ± 1.37	72.40 ± 0.11 (58.28)
Pusa Garima	136.02 ± 1.16	114.75 ± 0.77	48.55 ± 0.09 (44.15)
Pusa Gaurav	174.74 ± 0.64	171.02 ± 0.81	63.47 ± 0.13 (52.79)
Suryakiran	153.48 ± 2.08	167.93 ± 0.89	62.31 ± 0.11 (52.10)
Krishna	78.30 ± 0.85	87.96 ± 0.99	32.74 ± 0.09 (34.89)
Shola	143.08 ± 1.76	132.86 ± 1.15	49.55 ± 0.14 (44.73)
Pusa Pitamber	17.63 ± 1.54	23.51 ± 0.47	11.59 ± 0.18 (19.89)
Lahar	21.92 ± 0.86	27.47 ± 0.62	12.77 ± 0.05 (20.93)
Ganga	11.32 ± 0.59	20.61 ± 0.49	8.51 ± 0.27 (16.95)
Raja Ram Mohan Roy	18.91 ± 0.38	25.43 ± 1.33	11.62 ± 0.16 (19.92)
Iceberg	5.21 ± 0.39	8.92 ± 0.36	4.45 ± 0.30 (12.17)
Mridula	7.53 ± 0.79	11.77 ± 0.47	5.36 ± 0.32 (13.38)
Shabnam	11.11 ± 0.93	17.81 ± 1.04	8.23 ± 0.09 (16.66)
Chingari	92.40 ± 0.64	99.77 ± 0.46	44.66 ± 0.14 (41.92)
Surkhab	164.41 ± 0.74	161.40 ± 1.15	61.68 ± 0.09 (51.73)
Pusa Abhishek	130.60 ± 0.98	129.10 ± 1.05	58.40 ± 0.07 (49.82)
SEm±	1.96	1.26	0.14
CD ( *p* ≤ 0.05)	5.50	3.54	0.38

Values in parentheses are arc sin transformed values.

**Table 3:** Linear correlation coefficients (r) between total anthocyanin content, antioxidant assays (FRAP, DPPH), and total phenolic content in petals of 50 rose varieties obtained by Pearson’s analysis.

Parameters	TAC	FRAP	DPPH	TPC
TAC	1	0.934 **	0.796 **	0.932 **
FRAP		1	0.921 **	0.988 **
DPPH			1	0.920 **
TPC				1

** Correlation is significant at the 0.01 level (2-tailed).

## Data Availability

The data is contained within the article.

## References

[1] Raymond O., Gouzy J., Just J., Badouin H., Verdenaud M., Lemainque A., Vergne P., Moja S., Choisne N., Pont C. (2018). The *Rosa* genome provides new insights into the domestication of modern roses. Nat. Genet..

[2] Szołtysik M., Kucharska A.Z., Sokół-Łętowska A., Dąbrowska A., Bobak Ł., Chrzanowska J. (2020). The effect of *Rosa spinosissima* fruits extract on lactic acid bacteria growth and other yoghurt parameters. Foods.

[3] Lu J., Zhang Q., Lang L., Jiang C., Wang X., Sun H. (2021). Integrated metabolome and transcriptome analysis of the anthocyanin biosynthetic pathway in relation to color mutation in miniature roses. BMC Plant Biol..

[4] Kumari P., Raju D.V.S., Prasad K.V., Singh K.P., Saha S., Arora A., Hossain F. (2017). Quantification and correlation of anthocyanin pigments and their antioxidant activities in rose (*Rosa* × *hybrida*) varieties. Indian J. Agric. Sci..

[5] Shafei M.N., Rakhshandah H., Boskabady M.H. (2010). Antitussive effect of *Rosa damascena* in guinea pigs. Iran. J. Pharm. Sci..

[6] Rezaie-Tavirani M., Fayazfar S., Heydari-Keshel S., Rezaee M.B., Zamanian-Azodi M., Rezaei-Tavirani M., Khodarahmi R. (2013). Effect of essential oil of *Rosa damascena* on human colon cancer cell line SW742. Gastroenterol. Hepatol. Bed. Bench..

[7] Kumari P., Raju D.V.S., Singh K.P., Prasad K.V., Panwar S. (2018). Characterization of phenolic compounds in petal extracts of rose. Indian J. Hortic..

[8] Enaru B., Drețcanu G., Pop T.D., Stǎnilǎ A., Diaconeasa Z. (2021). Anthocyanins: Factors affecting their stability and degradation. Antioxidants..

[9] Cai D., Li X., Chen J., Jiang X., Ma X., Sun J., Tian L., Vidyarthi S.K., Xu J., Pan Z. (2022). A comprehensive review on innovative and advanced stabilization approaches of anthocyanin by modifying structure and controlling environmental factors. Food Chem..

[10] Li D., Wang P., Luo Y., Zhao M., Chen F. (2017). Health benefits of anthocyanins and molecular mechanisms: Update from recent decade. Crit. Rev. Food Sci. Nutr..

[11] Qin G., Xiaojun M. (2013). Composition and antioxidant activity of anthocyanins isolated from Yunnan edible rose (An ning). Food Sci. Hum. Well..

[12] Lee J.H., Lee H.J., Choung M.G. (2011). Anthocyanin compositions and biological activities from the red petals of Korean edible rose (*Rosa hybrida* cv. Noblered). Food Chem..

[13] Yang H., Shin Y. (2017). Antioxidant compounds and activities of edible roses (*Rosa hybrida* spp.) from different cultivars grown in Korea. Appl. Biol. Chem..

[14] Lee M.H., Nam T.G., Lee I., Shin E.J., Han A.R., Lee P., Lee S.Y., Lim T.G. (2018). Skin anti-inflammatory activity of rose petal extract (*Rosa gallica*) through reduction of MAPK signaling pathway. Food Sci. Nutr..

[15] Ren G., Xue P., Sun X., Zhao G. (2018). Determination of the volatile and polyphenol constituents and the antimicrobial, antioxidant, and tyrosinase inhibitory activities of the bioactive compounds from the by-product of *Rosa rugosa* Thunb. var. plena Regal tea. BMC Complement. Altern. Med..

[16] Saati E.A., Pusparini A.D., Wachid M., Winarsih S. (2018). The anthocyanin pigment extract from red rose as antibacterial agent. Malays. J. Fund. Appl. Sci..

[17] Kumari P., Panwar S., Namita T., Kaushai S., Ullas P.S. (2017). Pigment profiling of flower crops: A review. Ecol. Environ. Conserv..

[18] Singleton V.L., Rossi J.A. (1965). Colorimetry of total phenolics with phosphomolybdic-phosphotungstic acid reagents. Am. J. Enol. Vitic..

[19] Benzie I.F., Strain J.J. (1994). The ferric reducing ability of plasma (FRAP) as a measure of “antioxidant power”: The FRAP assay. Anal. Biochem..

[20] Braca A., De Tommasi N., Di Bari L., Pizza C., Politi M., Morelli I. (2011). Antioxidant principles from *Bauhinia terapotensis*. J. Nat. Prod..

[21] Zheng J., Ding C., Wang L., Li G., Shi J., Li H., Wang H., Suo Y. (2011). Anthocyanins composition and antioxidant activity of wild *Lycium ruthenicum* Murr. from Qinghai-Tibet Plateau. Food Chem..

[22] SAS Institute Inc (2018). SAS® University Edition. Quick Start Guide for Students with Visual Impairments.

[23] Mudalige R.G., Kuehnle A.R., Amore T.D. (2003). Pigment distribution and epidermal cell shape in *Dendrobium* species and hybrids. Hort. Sci..

[24] Zheng J., Yu X., Maninder M., Xu B. (2018). Total phenolics and antioxidants profiles of commonly consumed edible flowers in China. Int. J. Food Prop..

[25] Roman I., Stănilă A., Stănilă S. (2013). Bioactive compounds and antioxidant activity of *Rosa canina* L. biotypes from spontaneous flora of Transylvania. Chem. Cent. J..

[26] Sadighara P., Gharibi S., Jafari A.M., Khaniki G.J., Salari S. (2012). The antioxidant and flavonoids contents of *Althaea officinalis* L. flowers based on their color. Avicenna. J. Phytomed..

[27] Suzan O.Y., Sezai E. (2011). Antibacterial and antioxidant activity of fruits of some rose species from Turkey. Rom. Biotechnol. Lett..

[28] Sayed E.S., Hamed A., Bazaid S.A., Shohayeb M.M. (2012). Total phenolics and antioxidant activity of defatted fresh taif rose, Saudi Arabia. Br. J. Pharm. Res..

[29] Zeng Y., Deng M., Lv Z., Peng Y. (2014). Evaluation of antioxidant activities of extracts from 19 Chinese edible flowers. SpringerPlus.

[30] Nowak R., Gawlik-Dziki U. (2007). Polyphenols of *Rosa* L. leaves extracts and their radical scavenging activity. Z. Naturforsch. C..

[31] Özgen M., Scheerens J.C., Reese R.N., Miller R.A. (2010). Total phenolic, anthocyanin contents and antioxidant capacity of selected elderberry (*Sambucus canadensis* L.) accessions. Pharmacogn. Mag..

[32] Căta A., Ştefănuţ M.N., Tănasie C., Pop R. (2010). Comparative analysis of bilberries alcoholic extracts regarding to anthocyanins content, total phenolics and antioxidant activity. Ovidius. Univ. Ann. Chem..

[33] Schmitzer V., Veberic R., Osterc G., Stampar F. (2010). Color and phenolic content changes during flower development in groundcover rose. J. Am. Soc. Hortic. Sci..

[34] Singh M., Singh L., Srivastava S.B.L. (2016). Combining ability analysis in Indian mustard (*Brassica juncea* L. Czern & Coss). J. Oilseeds Res..

[35] Eugster C.H., Märki-Fischer E. (1991). The chemistry of rose pigments. Angew. Chem. Int. Ed..

[36] Ludmila D., Victor D., Valery T., Natalya M. (2015). Rose flower petals: Rich source of anthocyanins. Res. J. Pharm. Biol. Chem. Sci..

[37] Jing P.U., Noriega V., Schwartz S.J., Giusti M.M. (2007). Effects of growing conditions on purple corncob (*Zea mays* L.) anthocyanins. J. Agric. Food Chem..

[38] da Silva F.L., Escribano-Bailón M.T., Alonso J.J.P., Rivas-Gonzalo J.C., Santos-Buelga C. (2007). Anthocyanin pigments in strawberry. LWT-Food Sci. Technol..

[39] Wan H., Yu C., Han Y., Guo X., Luo L., Pan H., Zheng T., Wang J., Cheng T., Zhang Q. (2019). Determination of flavonoids and carotenoids and their contributions to various colors of rose cultivars (*Rosa* spp.). Front. Plant Sci..

[40] Raymond O., Biolley J.P., Jay M. (1995). Fingerprinting the selection process of ancient roses by means of floral phenolic metabolism. Biochem. Syst. Ecol..

[41] Biolley J.P., Jay M., Viricel M.R. (1994). Flavonoid diversity and metabolism in 100 *Rosa* x *hybrida* cultivars. Phytochem..

